# Syphilitic Gummata in the Central Nervous System: A Narrative Review and Case Report about a Noteworthy Clinical Manifestation

**DOI:** 10.3390/microorganisms9050906

**Published:** 2021-04-23

**Authors:** Lennart Barthel, Susann Hetze, Sarah Teuber-Hanselmann, Valérie Chapot, Ulrich Sure

**Affiliations:** 1 Department of Neurosurgery, University Hospital of Essen, Hufelandstraße 55, 45147 Essen, Germany; Susann.Hetze@uk-essen.de (S.H.); Ulrich.Sure@uk-essen.de (U.S.); 2 Institute of Medical Psychology and Behavioral Immunobiology, University Hospital of Essen, Hufelandstraße 55, 45147 Essen, Germany; 3 Institute of Neuropathology, University Hospital of Essen, Hufelandstraße 55, 45147 Essen, Germany; Sarah.Teuber@uk-essen.de; 4 Institute of Medical Microbiology, University Hospital of Essen, Hufelandstraße 55, 45147 Essen, Germany; Valerie.Chapot@uk-essen.de

**Keywords:** neurosyphilis, syphilis, treponema pallidum, syphilitic gumma, central nervous system gumma, cerebral syphilitic gumma, brain syphilis

## Abstract

Infection with *Treponema pallidum* is on the rise. In this narrative literature review, we show that the incidence of rare manifestations of syphilis, such as intracerebral gummata, is increasing and should be considered in the differential diagnosis of intracerebral lesions. With the exemplary case that we present here, we aim to raise awareness of the resurgence of this disease, which should be considered in the differential diagnosis of intracerebral lesions, especially for patients who have a risk profile for syphilis, and serological testing for *T. pallidum* prior to surgery should be discussed in order to avoid an unnecessary operation.

## 1. Introduction

*Treponema pallidum* is a Gram-negative, spiral-shaped bacterium (spirochete) of the family Spirochaetaceae that causes syphilis in humans [1,2]. The clinical manifestations of syphilis are diverse and depend on the stage of the disease, which can cause difficulty in distinguishing syphilis from other pathologies. After the Second World War, the worldwide incidence of syphilis decreased because of the efforts of the World Health Organization’s global health programs and the central health policies of the former Soviet Union [3,4]. However, the incidence has been increasing in Eastern European and Western countries, particularly among men who have sex with men (MSM) [3,5]. Neurosyphilis can occur at any time after an initial infection [6,7]. On the basis of clinical examination and laboratory findings, its manifestations can be classified according to disease phase as early, early or late, and late [7]. Early-stage neurosyphilis can be asymptomatic, although the meninges are usually involved. Meningovascular involvement can be present in the early or late phase [7]. The late phase is characterized by general paresis or tabes dorsalis. Syphilitic gummata usually occur in tertiary syphilis, a stage in which blood vessels, the heart, and the nervous system are affected. However, syphilitic gummata are rare and therefore not integrated into the aforementioned neurosyphilis classification scheme. Although the incidence of syphilitic gummata is low, an increased number of patients with intracerebral gummata should be expected in the future due to the recent increase in the incidence of syphilis. To the best of our knowledge, there are no reliable data regarding the prevalence or incidence of neurosyphilitic gummata. We therefore conducted a literature review of articles concerning syphilitic gummata in the central nervous system that were published in the past 30 years. Our review confirms that the incidence of neurosyphilitic gummata is again on the rise. Additionally, we describe, as an example, the case of a 51-year-old man with newly diagnosed syphilis who presented with an intracerebral gumma that was initially suspected as a glioma.

## 2. Case Presentation

**Figure 1:**
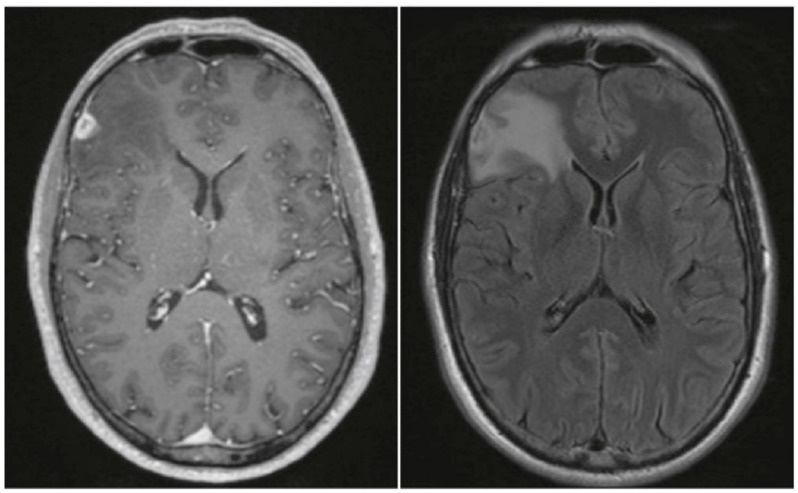
Magnetic resonance imaging of the neurocranium showed a small right frontolateral dural-based contrast-enhancing lesion measuring approximately 1.5 cm in diameter with perifocal oedema (**left**, T1-weighted sequence with contrast enhancement; **right**, fluid-attenuated inversion recovery (FLAIR) sequence).

**Figure 2:**
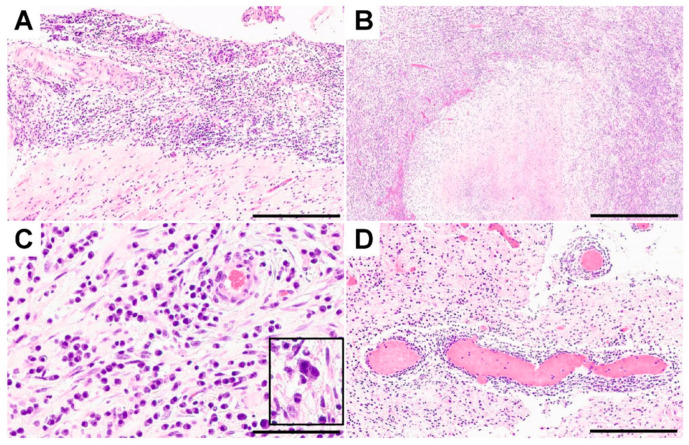
Fibrotic leptomeninges ((**A**) scale bar: 300 µm) and gliotic brain parenchyma ((**B**) scale bar: 500 µm) were observed, accompanied by foci of necrosis surrounded by densely packed inflammatory infiltrates comprised of lymphocytes, plasma cells, granulocytes, histiocytes, fibroblasts ((**C**) scale bar: 50 µm), and solitary multinucleated giant cells (inset in **C**). Leptomeningeal and intraparenchymal blood vessels showed perivascular inflammation without signs of vasculitis ((**D**) scale bar: 300 µm).

A 51-year-old man with leucocytic colitis presented in August 2016 with a one-month history of vertigo and blurry vision. He reported two episodes of tinnitus in February and April and a history of having sex with men. He was in a long-time monogamous relationship and his partner had no known previous infections. The patient had never been diagnosed with a sexually transmitted disease, nor had he received any serological testing for sexually transmitted diseases in the past. Active medications included antiandrogenic therapy for alopecia. A vestibular examination with Frenzel goggles showed spontaneous nystagmus. The remainder of his clinical examination was unremarkable. No skin or mucosal lesions were observed. Because of the inconspicuous clinical examination, no laboratory or instrumental testing was conducted, except imaging testing. Magnetic resonance imaging of the neurocranium showed a small right frontolateral dural-based contrast-enhancing lesion measuring approximately 1.5 cm in diameter with perifocal oedema (Figure 1). After consultation with our institutional interdisciplinary tumor board, surgery was recommended. During the operation, we noted that the lesion was supplied by cerebral vessels, had a firm leather-like consistency, and was not distinguishable from normal brain tissue—untypical for a glioma. Histological examination revealed fibrotic leptomeninges (Figure 2A) and infiltration of gliotic brain tissue by densely packed inflammatory cells surrounding necrotic foci (Figure 2B). Although these pathologies resembled granulomas to some extent, we did not identify any true granulomas. The infiltrates were embedded in collagenous tissue and comprised mainly plasma cells and lymphocytes accompanied by granulocytes, histiocytes, fibroblasts, epithelioid cells, and solitary multinucleated giant cells (Figure 2C). The blood vessels were surrounded by perivascular inflammatory cells but did not show any signs of vasculitis (i.e., necrosis or destruction and inflammation of the vessel walls) (Figure 2D). Bacterial and mycotic or mycobacterial pathogens were not detected in the histological specimen, nor by polymerase chain reaction (PCR), respectively. Additionally, immunohistochemical stainings for *Toxoplasma* species and *T. pallidum* were negative. Staining with antibodies against the glial fibrillary acidic protein (GFAP), p53, and IDH1-R132H were not suggestive of a brain tumor. Therefore, the histopathological diagnosis was necrotizing meningoencephalitis. Lumbar puncture for cerebrospinal fluid analysis was performed but did not show evidence of an infection. Although human immunodeficiency virus (HIV) infection was not detected, serum testing was positive for several treponemal tests (chemiluminescence assay (CLIA); *T. pallidum* hemagglutination assay (TPHA): 160,000 (reference, < 80 titer); rapid plasma regain (RPR) test for anti-cardiolipin antibodies: 8 (reference, < 1 titer)), as was cerebrospinal fluid testing (TPHA: 5000 (reference < 20 titer); RPR: 1 (reference, < 1 titer)). IgM antibodies for *T. pallidum* were positive (Treponema IgM: 51 IE/mL (reference < 20 IE/mL)). Prior to discharge on day 8 after surgery, the patient was referred to the dermatology department for a 21-day course of intravenous penicillin G (aqueous crystalline penicillin G, 24 million units per day, administered as 3–4 million units intravenous every 4 h for 10 days).

The *T. pallidum* serology parameters improved as well (CLIA: > 70; TPHA: 10,000; RPR: 4). Four months later, the patient had recovered completely. Serological parameters for *T. pallidum* were: CLIA: > 70; TPHA: 2560; RPR: < 1; IgM: 11 IE/mL.

## 3. Review

We have identified and extracted data from 49 neurosyphilitic gumma cases in addition to our case since 1990. An additional report describes six cases from the U.S. However, no conclusions can be drawn from the patient-specific information (report from 1992) [8]. In total, 8 of the 50 cases were women (mean age, 49.6 years; range, 37–65) and 42 were men (information about age was given in 39 cases: mean age, 45.5 years; range, 20–75). For two men, no age was reported; the age of one man was described as “in his 30s.” Among the 42 men, 5 were reported to be heterosexual (mean age, 46.6 years; range, 41–54). MSM was reported for four cases (mean age, 50 years; range, 42–61), and two of them were human immunodeficiency virus (HIV)-positive aged 42 and 46 years. Otherwise, 33 had no sexual preference reported. Overall, 14 of the men were HIV-positive (33.3%), and 2 of the 5 heterosexual reported men were HIV-positive. Among the men whose sexual preference was not reported, 14 were HIV-positive (42.42%). None of the women were reported to be HIV-positive, although HIV status was not reported for one case. Surgery was performed in 28 patients (56%), and 21 received conservative treatment only (42%). Information about surgery was not reported in one patient. In Table 1 are all 50 cases listed by location of the gumma, sex, MSM, age, HIV status, and country of the report. Many cerebral syphilitic gummata were reported in North America at the beginning of the 1990s. Since 2000, cases have been reported in Europe, and an increase in the overall number of reported cases began in 2016, predominately in China and Japan. In Table 2, it is shown how the patients clinically presented, whether the patients underwent an operation, which drug treatment was carried out, and the clinical outcome of the patients. As is typical of syphilis, the clinical symptoms varied widely, are difficult to divide into individual categories, and depend on the gumma’s localization. In most cases, the therapy regimen was carried out with IV penicillin, with differences in dosage and therapy duration (details in Table 2). Table 3 lists the diagnostic results (histological, CSF, and neuroimaging) for the corresponding cases. In the multitude of histological findings, inflammatory processes were demonstrated, often accompanied by increased vascularization. Strain analyzes for *T. pallidum* were not always positive. White blood cells were often present and increased or reactivated VDRL or a positive *T. pallidum* antibody index. Increased protein levels and pleocytosis were also often present. On imaging, syphilitic gummata presented mostly solid and contrasted medium, with perifocal edema and signs of inflammatory processes (details in Table 3).

## 4. Discussion

Although reports of intracerebral gummata are rare [9], the increasing reports of syphilis suggest that healthcare professionals will encounter an increasing number of cerebral manifestations, as shown by our review. With this case report and review, we aim to raise awareness of the resurgence of this well-known, but mostly historical, disease that should be considered in the differential diagnosis of intracerebral lesions.

## Figures and Tables

**Table 1:** Listed are all identified cases between the years 1990 and 2021. Ordered by years of the report. The location of the syphilitic gumma, sex, men who have sex with men (MSM), age, human immunodeficiency virus (HIV) status, and country of the report are presented. If no information was available for one of the categories, the table’s casket was left blank. (Abbreviations: f, feminine; HIV, human immunodeficiency virus; m, masculine.).

# of Case	Location	Sex	MSM	Age	HIV	Year of Report	Country	Ref.
1	Frontal lobe; thoracic vertebrae	f		37	no	2021	U.S.	[9]
2	Frontal lobe	m		45		2019	China	[10]
3	Thoracic medullary Th10; cauda equina at the 3/4 lumbar level	m		“In his 30s”	no	2019	Australia	[11]
4	Multiple loci cerebellar and medullary	m		22	no	2019	China	[12]
5	Frontal and temporal lobe	m		47	no	2019	Japan	[13]
6	Thoracal intra-/extramedullary T8 level	f		45	no	2019	China	[14]
7	Frontal lobe	m		37		2018	China	[15]
8	Frontal lobe	m		62		2018	China	[15]
9	Frontal lobe	m		66		2018	China	[15]
10	Temporal lobe	m		62	no	2018	Japan	[16]
11	Temporal lobe	m	no	44	yes	2018	Japan	[17]
12	Temporal	m		36	no	2018	Japan	[18]
13	Frontal lobe	f		50		2018	Japan	[19]
14	Medullary L4	m		61		2017	China	[20]
15	Frontal lobe	m		62		2017	China	[21]
16	Brain stem	m	no	41	no	2017	China	[22]
17	Optic nerve	m		36	yes	2016	U.S.	[23]
18	Parietal lobe	m		56	no	2016	China	[24]
19	Intramedullary C5 level	f		65	no	2016	China	[25]
20	Frontal lobe	m		21	yes	2016	Japan	[26]
21	Occipital lobe	m		53		2016	Greece	[27]
22	Frontal lobe	m	yes	51	no	2016	Germany	This case
23	Parietal lobe	m		40	yes	2014	Switzerland	[28]
24	Frontal lobe	f		59	no	2013	South Korea	[29]
25	Parietal	m		38	no	2013	China	[30]
26	Pons	m		26	yes	2012	Brazil	[31]
27	Cavernous sinus	f		62	no	2011	South Africa	[32]
28	Basal ganglia; temporal lobe	m		45	no	2011	China	[33]
29	Cerebrum	f		40	no	2009	South Korea	[34]
30	Occipital lobe	m	yes	61	no	2008	U.S.	[35]
31	Frontal lobe	m		20	yes	2008	Brazil	[36]
32	Frontal lobe	m	yes	46	yes	2008	Canada	[37]
33	Temporal lobe	m	no	43	no	2008	Australia	[38]
34	Parietal lobe	m		42	yes	2007	Portugal	[39]
35	Parietotemporal	m	no	54		2007	U.S.	[40]
36	Frontal lobe	m	yes	42	yes	2005	Belgium	[41]
37	Corpus callosum	m		42	no	2005	U.S.	[42]
38	Pons and middle cerebellar peduncle	m	no	51	yes	2003	Brazil	[43]
39	Temporo-parietal	m		42	yes	2002	Israel	[44]
40	Temporo-occipital	m		75		1999	Japan	[45]
41	Frontal lobe	m		47	no	1996	U.S.	[46]
42	Optic tract, temporal lobe	f		39	no	1995	Japan	[47]
43	Cerebellar, temporal lobe	m		51		1995	Japan	[48]
44	Parieto-occipital, cerebellum	m		29	yes	1994	U.S.	[49]
45	Pontomesencephalic region; choroidal fissure	m		69	no	1994	U.S.	[50]
46	“Cerebral”	m			yes	1992	U.S.	[51]
47	“Cerebral”	m			yes	1992	U.S.	[51]
48	Tuber cinereum	m		37		1991	U.S.	[52]
49	Midbrain and thalamus	m		27		1991	U.S.	[53]
50	Optic nerve	m		68		1990	U.S.	[54]

**Table 2:** Listed are all identified cases between the years 1990 and 2021. Ordered by years of the report (as in Table 1). Shown are how the patients clinically presented, whether an operation was carried out, which drug treatment was carried out, and the outcome. (Abbreviations: IM, intramuscular injection; IV, intravenous therapy.).

# of Case	Clinical Appearance	Surgery	Treatment	Outcome	Ref.
1	Seizures, left-sided weakness, urinary incontinence, mild photophobia, ataxia, and headache.	Yes	Benzathine penicillin G daily; dexamethasone.		[9]
2	Headache and left-sided weakness.	Yes	14-day course of IV penicillin (2.5 million U administered every 4 h) followed by a three-week course of IM of benzathine penicillin (2.4 million U administered once per week).	Myodynamia of the left limbs gradually improved.	[10]
3	Rapidly progressive right lower limb monoparesis associated with sphincter and erectile dysfunction; impaired sensation in the right leg, with reduced anal tone and saddle anesthesia.	Yes	IV benzylpenicillin.	Near complete neurological recovery after three months.	[11]
4	Progressive right lower limb weakness with tremor, and headache.	No	IV penicillin G at 24 million units daily divided into six doses was given for a total of 21 days, along with three weekly IM of benzathine penicillin G (BPG, 2.4 million units); oral prednisolone (40 mg) was also prescribed 24 h before the start of penicillin for three days.	Complete neurological recovery after three weeks.	[12]
5	Generalized tonic-clonic seizures; syphilitic roseola was observed on the skin in the hypogastric region.	no	Amoxicillin per os 1500 mg/day followed by penicillin G IV 24 × 10^6^ U for 14 days.	Completely recovered.	[13]
6	Revealed muscle strength of grade 3/5 in right lower extremities and loss of superficial sensation of grade 3/10 below the umbilicus on the left side; grade 3 ankle and knee hyperreflexia on the right side; positive Babinski sign and Chaddock sign.	yes	Penicillin G (IV 19.6 million U/day, and 4.9 million U QID) for 14 days, then IM benzathine penicillin G (2.4 million U QW) for 21 days.	Completely recovered after three months.	[14]
7	Dizziness for ~15 days.	no	14-day IV penicillin (2.4 million U every 4 h) followed by three weeks of IM benzathine penicillin (2.4 million U, once per week).	Complete recovery after four months.	[15]
8	Speech arrest for 10 h, clear consciousness.	yes		Complete recovery after six months.	[15]
9	Decreasing right eye vision and headache since ~50 days ago.	yes			[15]
10	Headache.	yes			[16]
11	Severe headache, nausea, and vomiting.	yes	14 days of IV ceftriaxone 2 g every 24 h.	Completely recovered.	[17]
12	Hearing loss in right ear and right-side facial paralysis since two weeks ago.	no	“2015 U.K. national guidelines”.	After two weeks: facial nerve palsy improved markedly; hearing loss improved gradually.	[18]
13	Headache, right-sided hearing loss, tinnitus, and vertigo since three months ago.	no	IV penicillin G.		[19]
14	Worsening pain and numbness in both lower legs for four weeks, started one year ago.	yes			[20]
15	Speech disturbance and a mild headache since 10 days ago.	yes	Penicillin G IV daily (24 million units for 14 days), followed by IM benzathine penicillin G (2.4 million units three-weekly).	“Improving gradually”.	[21]
16	Headache, swallowing difficulties, and dysarthria for four months, and vomiting for a month and a half; progressive right facial and neck numbness for two years; dysarthria; right Babinski sign positive.	no	IV penicillin G (24 million units per day).	Complete recovery after two months.	[22]
17	Decreased vision, left eye.	no	14 days of IV penicillin.	Vision improved.	[23]
18	Mild headache; twitch and right limb sustained shaking.	yes	IV penicillin G (24 million units per day for 12 days), followed by IM benzathine penicillin G (2.4 million units three-weekly).		[24]
19	Paresthesia in both upper extremities and weakness; two-month history of neck–shoulder–back pain.	yes	IV penicillin G (24 million units/day) for 14 days.	After 29 months: pain completely relieved; sensorimotor dysfunctions partially improved; sensory disturbance remained; muscle strength legs improved.	[25]
20	2 h loss of consciousness; prior: uncomfortable feeling at the back of his head and neck and eye fatigue that lasted for one week.	no	IV benzylpenicillin (24 million units/day) for 14 consecutive days.	Complete recovery after two months.	[26]
21	Rapidly deteriorating gait disorder.	yes	“Per os penicillin”.	Complete recovery.	[27]
22	One-month history of vertigo and blurry vision; two episodes of tinnitus.	yes	IV penicillin G (24 million units per day, 3–4 million units every 4 h) for 10 days.	Complete recovery after four months.	This case
23	Persistent fatigue, excessive sweating and pain in the right thorax, slight paresthesia of the right hand, and headaches.	yes	IV penicillin G (6 × 4 Mio U/day) for 14 days.		[28]
24	Speech disturbance/dysarthria.	yes	Ceftriaxone IV daily dose of 2 g for 14 days.	Completely recovered after 15 months.	[29]
25	Headache and emesis for 15 days.	yes		“Symptoms are improving gradually”.	[30]
26	Four-month history of fever, weight loss, dizziness, diarrhea, tremors, and paresthesia, disorientation, pyramidal and extrapyramidal symptoms, and multiple cutaneous non-pruriginous cicatricial lesions affecting the chest and inferior limbs.	no	IV crystalline penicillin (24 × 10^6^ units/day) for 14 days.	“Significant clinical improvement”.	[31]
27	Two-week history of painful ophthalmoplegia and a complete left ptosis, signs of cavernous sinus syndrome, with left sided III, IV, V1, V2, and VI cranial nerve palsies.	no	IV crystalline penicillin G (24 million units per day) for two weeks, followed by 2.4 million units of IM penicillin administered weekly for three weeks (total 7.2 million units).	Cranial nerves: After three weeks, full recovery in III, IV, V1, and V2, and partial recovery in cranial nerve VI.	[32]
28	Eight-day history of right-sided vision loss, slurred speech, incoordination of his right arm and leg, and imbalance ad admission.	yes	IV penicillin G 14 days, followed by three-weekly IM shots of benzathine penicillin G (2.4 million units).	One-month follow-up: gait and use of right hand improved dramatically; right-sided visual loss persisted.	[33]
29	Three- to four-month history of headache.	yes	IV penicillin G daily (24 × 10^6^ U) for 10 days.	Complete recovery.	[34]
30	Two-month history of progressively worsening altered mental status and intermittent seizures characterized by the déjà vu phenomena; left homonymous hemianopsia.	yes			[35]
31	Single episode of a tonic-clonic seizure.	yes	IV crystalline penicillin G (24 million units) daily for 14 days.		[36]
32	Eight-week history of left frontal headaches.	no	“Intravenous penicillin”.	No further symptoms.	[37]
33	Two-month history of worsening generalized headaches, nausea, and peculiar speech.	yes	“Intravenous penicillin”.	Full clinical recovery.	[38]
34	Generalized seizure.	no	IV penicillin G; after 10 days, the patient refused further treatment.	After two-month asymptomatic period, follow-up MRI: residual focal contrast enhancement, marked reduction of perilesional edema, and normal signal on diffusion weighted imaging (DWI).	[39]
35	“Altered mental status,” rhabdomyolysis, and hypernatremia; confused, disoriented, and agitated; “speech was incoherent, his mood anxious, and his affect inappropriate”.	no		The patient died eight days after admission; the diagnosis of neurosyphilitic gumma was made post-mortem.	[40]
36	Fever, headache for two and a half months, and hearing loss since one week before	no	IV penicillin G and ampicillin for three weeks	Complete recovery after three weeks	[41]
37	Frequent falling, visual hallucinations, headaches, diminished appetite, and prominent weight loss over a period of several months; cachectic and minimally interactive; prominent abulia, psychomotor retardation, and tremulousness of bilateral upper extremities; diminished proprioception with a shuffling gait	yes		Patient died during hospital stay	[42]
38	Three-month history of progressive visual decline in the right eye	no	IV penicillin (24 million units/day) for 21 days	Lost to follow-up	[43]
39	Grand mal seizure; ataxia	yes	Treatment for tuberculosis	Died after three weeks; post-mortem diagnosis of syphilis	[44]
40	10-day history of headache	yes	IV penicillin G: Skin eruption; switch to oral erythromycin for 28 days	Recovery	[45]
41	Generalized seizure	no	IV phenytoin and high-dose penicillin G for 21 days	Recovery	[46]
42	Visual impairment worsening rapidly over one week; left upper quadrantanopsia, diplopia, slight hemiparesis, and hypesthesia on the left side; bilateral optic atrophy	yes	IV penicillin G (24 × 10^6^ U/day IV) plus probenecid for 14 days, followed by procaine penicillin G (2.4 × 10^6^ U/week IM)	Rigidity in left upper extremity resolved; left upper homonymous quadrantanopsia remained	[47]
43	Four-month history of headache; diplopia for three months; vertigo; cerebellar ataxia; abducens nerve paresis	yes	IV penicillin G (12 × 10^6^ U/day) for 10 days (total 120 × 10^6^ U)	Cerebellar ataxia gradually improved; right abducens nerve paresis remained unchanged	[48]
44	Three years before: history of right-sided weakness for six months, changes in mental status for three months, one seizure one week before admission, problems with swallowing for one week; at admission: disorientation, tremoulus, and poor memory	no	Cefotaxime 2 g IV every 6 h for 11 days, followed by IV penicillin G (12 × 10^6^ U/day) for 10 days; subsequently, amphotericin B and ceftazidime (because of progressive neurological deterioration)	None of the lesions resolved with treatment; four days before death, occlusive hydrocephalus	[49]
45	Intermittent diplopia, slurred speech, right-sided weakness, and gait unsteadiness; after therapy (five months later): Mild worsening of the right-sided hemiparesis	no	Allergy to penicillin: two-week IV ceftriaxone (1 g/day), followed by 30 days of oral doxycycline (100 mg twice a day); second treatment five months after initial treatment: skin tests for penicillin allergy; subsequently, 21 days of IV penicillin (4 million U every 4 h)	Five months after initial therapy: Mild worsening of hemiparesis; at follow-up six months after last therapy (penicillin): Remained neurologically stable; findings on a repeated lumbar puncture were normal	[50]
46	Seizure disorder	yes	“High-dose” IV penicillin		[51]
47	Seizure disorder	no	“High-dose” IV penicillin		[51]
48	Headache for three months; mild ataxia, intermittent low-grade fever, skin rashes, cervical lymph node enlargement, conjunctivitis, and progressively decreased libido		Initial diagnosis sarcoidosis: prednisone; after second lumbar puncture and diagnosis of syphilis: IV penicillin G (24 million units per day) for 10 days.	Three months after therapy: Neurological examination was normal; left hemi-ataxia resolved	[52]
49	Dorsal midbrain syndrome, cognitive dysfunction, and a left peripheral seventh nerve palsy	no	“Intravenous penicillin”		[53]
50	Right eye vision suddenly became “totally black,” cleared totally within 10–12 s; edema of the right optic nerve with dilated vessels on its surface and a bit of hemorrhage around the papilla; less than one month later: transient obscurations in the right eye, floaters in the left eye, photophobic, and persistent blurring left eye	no	IV penicillin G (20 million U/day) for 10 days	Transient obscurations stopped completely during hospital stay; after five months: right optic disc appeared notably improved; blind spot sizes within normal limits in both eyes; iritis and vitritis of left eye improved	[54]

**Table 3:** Listed are all identified cases between the years 1990 and 2021. Ordered by years of the report (as in Table 1). Shown are the histological, CSF, and neuroimaging findings. (Abbreviations: CD, cluster of differentiation; CSF, cerebrospinal fluid; CT, computed tomography; DNA, deoxyribonucleic acid; DWI, diffusion-weighted magnetic resonance imaging; FLAIR, fluid-attenuated inversion recovery; FTA-ABS, fluorescent treponemal antibody absorption; GFAP, glial fibrillary acidic protein; HE, hematoxylin and eosin; HHV, human herpesvirus; IgG, immunoglobulin G; MRI, magnetic resonance imaging; SPECT, single-photon emission computed tomography; MBP, mannose-binding lectin; NF, neurofilament; PAS, periodic acid–Schiff; PCR, polymerase chain reaction; RPR, rapid plasma regain; TPHA, *Treponema pallidum* hemagglutination assay; TPLA, *Treponema pallidum* latex agglutination; TPPA, *Treponema pallidum* particle agglutination assay; TRUST, toluidine red unheated serum test; VDRL, Venereal Disease Research Laboratory).

# of Case	Histology	CSF	Neuroimaging	Ref.
1	Focal chronic dural inflammation and a reactive neocortex with chronic inflammation and rare spirochetes.		MRI: cerebral edema of the frontoparietal lobes; nodular contrast enhancement (T1).	[9]
2	Large quantity of inflammatory cell infiltration containing lymphocytes, neutrophils, and necrosis.	Routine examination, biochemical indexes: normal; TPPA: positive.	Edema around the lesion in MRI and CT. CT: low-density lesion; homogeneous enhancement with contrast. MRI: isointensity on T1; long T2 nodular signal shadow; somewhat higher T2-FLAIR signal; high signal in diffusion-weighted imaging.	[10]
3	Initial Warthin–Starry staining for spirochetes, Ziehl–Neelsen staining for atypical bacteria, and periodic acid-Schiff staining for fungi were negative; retrospective immunoperoxidase stains returned positive and revealed scattered spirochetes.		MRI: lobulated contrast enhancing intramedullary mass at level T10.	[11]
4		White blood cells: 84 cells/mL; total protein level: 2.08 g/L; glucose level: 2.95 mmol/L; TRUST: positive (1:4).	MRI: multiple dural-based enhancing masses; irregular ring-enhancing lesion, central hypointense surrounding edema; enhanced nodules: homogeneous-enhancing or ring-enhancing.	[12]
5		Cell count: 199/mL; glucose: 61 mg/dL; protein: 116 mg/dL; positive TPHA and FTA-ABS—immunoglobulin G.	MRI: multiple mass lesions, enhanced and adjacent to the dura, left cerebral hemisphere.	[13]
6	Granulomatous inflammation with small areas of caseous necrosis, multinucleated giant cells infiltration, surrounded by large numbers of lymphocytes and small numbers of neutrophils; swelling and hyperplasia of some vascular endothelial cells with massive infiltration of lymphocytes and plasma cells around the blood vessels; immunohistochemistry: immunopositivity with glial fibrillary acidic protein, myelin basic protein, neurofilament protein, CD3, CD45RO, and CD68, but was negative for periodic acid-Schiff and CD56; acid-fast staining: negative; further Warthin–Starry staining confirmed spirochetes.		MRI: irregular nodule at T8 level intradural–extramedullary and intramedullary, slightly hyperintense (T1), heterogeneously hyperintense signal (T2), significantly and homogeneously enhanced with contrast.	[14]
7		Protein: 97.3 mg/dL; white blood cells: 84 × 10^6^/L; RPR: positive; TPPA: positive.	MRI: slightly abnormal lamellar and longer T1, T2 signal shadow; contrast enhancement: lesion patchy enhancement, adjacent meninges slightly thickened and enhanced.	[15]
8	HE staining: necrotic with infiltration of inflammatory cells, glial proliferation in the periphery; GFAP staining: small amount of glial proliferation around necrotic foci; Ki67 staining: higher proliferative activity around the necrotic lesions; P53 staining: negative peripheral P53.		MRI: slightly long T1 and a long T2 nodular signal shadow left cerebral falx, slightly high T2- FLAIR and DWI signal; edema. CT: low-density-area left frontal lobe, ventricular compression.	[15]
9	Argyrophilic staining: negative; HE staining: necrotic with infiltration of inflammatory cells, glial proliferation in the periphery; GFAP staining: small amount of glial proliferation around necrotic foci; Ki67 staining: higher proliferative activity around the necrotic lesions; P53 staining: negative peripheral P53.	TPPA: positive; RPR: positive.	MRI: irregular clumping, high-signal mixed with low-signal foci frontal lobe, unclear border, surrounded by a large, low-signal shadow, ventricle re-compressed.	[15]
10	Immunohistochemical staining revealed numerous spirochetes.	2.2-fold higher RPR levels.	Contrast-enhanced T1-weighted, fluid-attenuated inversion recovery image reveal ring-enhanced lesion with substantial edema.	[16]
11	Nonspecific inflammatory granuloma with central necrosis; *T. pallidum* immunohistochemical stain: clearly stained as helical-shaped in the granuloma specimen (two different *T. pallidum*-specific PCR (targeting polA and TpN47) for homogenized specimens were positive; *T. pallidum* DNA was identified.	Cells: 12/mm^3^; glucose: 98 mg/dL; protein: 90 mg/dL; TPLA: negative; RPR: negative.	MRI: nodule with ring enhancement; high-intensity area in T2; SPECT: weak uptake both in early and late phase; high retention index of 0.86.	[17]
12		142 cells/µL (96% lymphocytes); glucose: 60 mg/dL; total protein: 64 mg/dL; RPR titer: 1:2.4; *Treponema pallidum* latex agglutination titer: 1:53.4.; fluorescent treponemal antibody absorption: 2+ positive.	MRI: nodulus-enhanced temporal on T1, hyperintense on T2; enhanced vestibulo-cochlear nerve and facial nerve T1.	[18]
13		Fluorescent treponemal antibody absorption: increased (1:514.5).	MRI: enhancing mass; iso to slightly hyperintense lesion (T1). CT: iso-attenuating lesion; mild enhancement, surrounding edema; hypointense with surrounding edema (T2*); hypointensity at cortex with surrounding hyperintensity (DWI; postcontrast T1: heterogeneous enhancement). CT perfusion: no increase in cerebral blood volume.	[19]
14	Degenerative necrotic tissues and fibrous connective tissues with occasional perivascular infiltration by lymphocytes.		MRI: narrowing of the disc space at L4–5, mass behind vertebral body. CT: “Extensive wormy appearance”.	[20]
15	Severe inflammation and putrescence formation with a large quantity of inflammatory cell infiltration (mainly of the lymphocytes and plasma cells).	RPR and *Treponema pallidum* particle agglutination test: positive; RPR titer: 1:8.	MRI: irregular-enhancing lesion with extensive edema. CT: lesion frontal lobe with severe edema.	[21]
16		50 cells/µL (80% lymphocytes, 20% monocytes); total protein level: 0.29 g/L; chloride concentration: 126.1 mmol/L; RPR + VDRL: negative.	MRI: hyperintense gadolinium-enhanced T1-weighted regions in the brainstem.	[22]
17			MRI: enhancement of the left optic nerve.	[23]
18	Severe inflammation; putrescence and abscess formation; large quantity of inflammatory cell infiltration (mainly of the plasma cells); Warthin–Starry staining: no spirochetes.	Protein level: 0.72 g/L; RPR: negative; TPPA: positive; Spirochetes: not detected.	MRI: mass lesion.	[24]
19	Granuloma with fibrous hyperplasia; large quantities of inflammatory cell infiltration; immunohistochemistry positive for GFAP, MBP, NF, CD3, and CD45RO; CD68 immunonegative: PAS and CD56; acid-fast staining: negative; Warthin–Starry staining: spirochete-positive.	VDRL: 1:16 dilution; TPPA assay: positive; very few cells; protein: 29 mg/dL; glucose: 57 mg/dL.	MRI: intramedullary nodule; isointense (T1); hyperintense with isointense center, perilesional oedema (T2); lesion enhanced after contrast.	[25]
20		Leukocyte count: 35 cells/mL (2 neutrophils/mL, 33 lymphocytes/mL); total protein level: 30 mg/dL; glucose: 59 mg/dL; RPR titer: 1:<1; TPHA titer: 1:160; fluorescent treponemal antibody absorption titer: 1:32.	CT: hypodense lesion. MRI: hypointense lesion by gadolinium-enhanced (T1), hyperintense (T2); extensive edema.	[26]
21	Necrotic area (star) with extensive peripheral granulomatous tissue (arrowhead).		Two lesions. MRI: low-signal (T1); ring-shaped enhancement and blurry borders (contrast enhanced); diffuse high-signal lesion (edema), low-signal, high-signal border (T2). CT: hypointense lesion, moderate edema.	[27]
22	Immunohistochemical stains: *Toxoplasma* spp.- and *T. pallidum*-negative; staining with antibodies to GFAP, p53, and IDH1-R132H negative.		Contrast-enhancing lesion with perifocal edema, contrast enhancement.	This case
23	Epithelioid cell macrophages and plasma cells without evidence of a pathogen.	TPHA: CSF/serum index negative.	MRI: mass lesion.	[28]
24	Chronic inflammation; Warthin–Starry: no spirochetes; necrotic material infiltrated predominantly with plasma cells; peri-vascular region with fibrosis contained lymphocytes and plasma cells; parenchymal infiltration of lymphocytes and plasma cells in the gumma.	White cells: 0/dL; erythrocytes: 1/mm^2^; glucose: 74 mg/dL; protein: 16.8 mg/dL; VDRL test: negative; *T. pallidum* (PCR): negative; FTA-ABS IgG: reactive.	MRI: irregular-enhancing mass, central necrosis; edema. CT: mass-like lesion; severe swelling.	[29]
25	Vascular intimal hyperplasia and large quantities of inflammatory cell infiltration; Warthin–Starry stain: *T. pallidum*-positive.	Protein: 0.468 g/L; chloride: 133.2 mmol/L; glucose: “normal”; lactate: “normal”; “no demonstrable *T. pallidum*”	MRI: irregular nodulus; hypointense (T1); hyperintense (T2) with meningeal thickening; edema. Contrast: enhancing ring.	[30]
26		“Aseptic meningitis” (lymphocytic pleocytosis, elevated protein, and normal glucose levels)	MRI: Pontine lesion; isointense to gray matter (T1WI); hyperintense on (T2WI and flair); no contrast enhancement.	[31]
27		VDRL: positive; FTA: positive; Ziehl–Neelsen stain: positive; protein: 0.37 g/L; glucose: 4.0 mmol/L; polymorphonuclear cells: 0 cells/mm^3^; lymphocytes: 8 cells/mm^3^; erythrocytes: 6 cells/mm^3^.	MRI: Left sphenoid wing dural-based enhancing mass.	[32]
28	Non-monoclonal, perivascular inflammatory infiltrates; no viral inclusions, granuloma or inclusions.	White blood cells: 18; red blood cells: 350; VDRL: positive.	MRI: lesions with isointense signaling (T1); surrounding increased signal and mass effect (T2); CT (with contrast): homogeneously enhancing lesions.	[33]
29	Central portion of the mass: necrotic material infiltrated with eosinophils; peripheral portion: fibrotic0contained lymphocytes and plasma cells; Warthin–Starry staining: spirochete-positive.	Red blood cells: 0 cells/mm^3^; white blood cells: 3 cells/mm^3^; glucose: 65 mg/dL; protein level: 47.0 mg/dL; VDRL, FTA-ABS; IgG: positive.	MRI: mass with an ill-defined margin, accompanied with severe swelling; central portion hypointense, peripheral isointense (T1); central portion hyperintense, peripheral portion isointense (T2); enhancement peripheral portion; no enhancement in the central portion (T1 contrast); high-signal intensity in the central portion (DWI).	[34]
30	Atypical polymorphic inflammatory infiltrate with intralesional spirochetes; fluorescein immunostaining: consistent with syphilitic gumma.	Glucose, protein, and cell counts: “Normal”; VDRL: nonreactive.	MRI: Isodense lesion (T1); isointense, extensive perilesional edema (T2); enhanced uniformly in contrast.	[35]
31	Granulomatosis with inflammatory infiltration; reactional gliosis, especially in the perivascular space; Ziehl–Nielsen and Groccot staining: negative; *T. pallidum*: positive.	White blood cells: 2/mm^3^; protein: 26 mg/dL; glucose: 69 mg/dL; VDRL: negative.	MRI: lesion enhanced in contrast.	[36]
32	Intense lymphoproliferative infiltrates of plasma cells, T lymphocytes, and B-cell infiltrates; PCR: positive for *T. pallidum*; Warthin–Starry stain: positive spirochaetes.	VDRL: reactive.	CT: left frontal lobe mass.	[37]
33	Necrotizing inflammatory mass, intense granulation; layered appearance: outer layer of reactive glial tissue, middle layer of granulation tissue containing lymphocytes, neutrophils, and plasma cells, and inner layer of necrosis; PCR: *T. pallidum* positive.	Aseptic meningitis with mononuclear pleocytosis; elevated protein with a low glucose CSF/serum ratio; RPR: positive.	MRI and CT: irregularly enhancing lesion with a central hypointense area, extensive surrounding edema.	[38]
34		“Normal cytology, glucose, and protein levels”; polymerase chain reactions for herpes simplex, cytomegalovirus, HHV6, and enteroviruses: negative; anti-treponemal antibodies: positive; VDRL: positive.	MRI: cortical lesion, isointense (T1, T2, fluid-attenuated inversion); edema; nodular and meningeal enhancement (contrast); restricted diffusion modulus and meningeal-based tail (DWI).	[39]
35	Syphilitic gumma on postmortem neuropathologic examination: well-defined, round, rubbery, gray-tan, 4 cm maximal diameter mass, with adjoining diffuse edema; diffuse thickening of leptomeninges and infiltration with lymphocytes and plasma cells peri-vascularly, and histiocytes within leptomeninges; Warthin–Starry and modified Steiner stain did not demonstrate treponemas.		CT: without contrast left middle cerebral artery infarct, with edema and mass effect.	[40]
36		White blood cell count: 1010/µL (64% polymorphonuclear leukocytes); hypoglycorrhachia: 16 mg/dL; protein level: 0.17 g/dL; lactate level: 50 mg/dL; IgG index: 1.27; 16 oligoclonal bands; anti-treponemal antibodies: positive; VDRL: positive.	MRI: vasogenic edema; enhancement of gumma (contrast), edema.	[41]
37	Necrotic areas with extensive mixed inflammation, consisting of lymphocytes, plasma cells, neutrophils, and focal collagen deposits; inflammation was also present in several midsized arteries, with extensive infiltration by macrophages and severe narrowing of the lumens.		MRI: heterogeneous enhancement of partially cystic midline butterfly-shaped intra- and extra-axial mass; edema; butterfly midline lesion with mild surrounding edema (postcontrast T1); necrotic regions within the lesion and surrounding inflammation and edema (FLAIR); no hyperintensity in the lesion (DWI). CT: heterogeneous perifalcine mass extending from the corpus callosum bilaterally into the subcortical regions of the frontal lobes with considerable mass effect.	[42]
38		26 cells/mm^3^ (94% lymphocytes); protein level: 106 mg/dL; glucose level: 65 mg/dL; VDRL and FTA-ABS: reactive.	MRI: contrast-enhancing lesions.	[43]
39	Non-specific encephalitis.	Autopsy: rich lymphocytes and plasma cells around blood vessels at the border of the gummas.	CT: three-ringed space-occupying lesions, surrounding edema.	[44]
40	Granulomatous inflammation; necrosis, fibrosis, and infiltration of a large number of lymphocytes and plasma cells.	TPHA: positive.	MRI: hypointense lesion (T1, T2), and strongly enhanced (contrast). CT: irregular low-density, ring-like enhancement (contrast).	[45]
41		Protein level: 117 mg/dL; glucose level: 69 mg/dL; white blood cells: 11 per mm^3^ (82% lymphocytes and 18% polymorphonuclear cells); VDRL: positive.	MRI: “abnormal signal” (T2); multifocal contrast enhancement.	[46]
42	Necrotic center, surrounded by a layer of granulation tissue infiltrated with proliferating fibroblasts, variable numbers of lymphocytes, macrophages, and histiocytes, and many newly formed small blood vessels; perivascular lymphocytes and histiocytes.		CT: Small ring-like enhanced mass with a surrounding low-density area. MRI: low-signal-intensity basal ganglia (T1); small ring-like enhancement in the vicinity of the right optic nerve (contrast); high-signal intensity surrounding the lesion (T2).	[47]
43	Epithelioid granuloma; central caseating necrosis, plasma cell infiltration; destruction of the tunica media of small arteries embedded in the lesion.	TPHA: positive.	CT: ambiguous hypodense lesions; oval homogeneously contrast-enhanced mass lesion attached to the dura mater right temporal. MRI: low-intensity lesions in right cerebellar hemisphere, right middle cerebellar peduncle, and right temporal lobe (T1); and high intensity in T2, homogeneously enhanced (contrast).	[48]
44	Postmortem: lesions with rubbery greenish core, surrounded by darker area; necrosis with marked inflammatory exudate (lymphocytes and plasma); multinucleated giant cells; silver staining with modified Steiner stain: spirochetal forms; PCR of coded specimens: syphilis (confirmed with DNA hybridization).	Three years ago: white blood cells: 4/mm^3^ (lymphocytes); erythrocytes: 3/mm^3^; protein level: 140 mg/dL; glucose level: 40 mg/dL; VDRL: negative. Last admission: white blood cells: 12/mm^3^ (11 lymphocytes); erythrocytes: 0; glucose level: 50 mg/dL; protein level: 178 mg/dL; VDRL: positive.	CT: multiple ring-enhancing lesions, left frontal.	[49]
45		Protein level: 23 mg/dL; glucose level: 86 mg/dL; leukocytes: 28/mm^3^; erythrocytes: 2/mm^3^; VDRL: not recorded. Second CSF sampling after one month: protein level: 82 mg/dL; glucose level: 60 mg/dL; leukocytes: 38/mm^3^ (99% lymphocytes); VDRL: positive. Third lumbar puncture after five months (after therapy): protein level: 40 mg/dL; glucose level: 55 mg/dL; leukocytes: 13/mm^3^ (86% lymphocytes); VDRL: positive.	MRI: contrast-enhancing lesions (T1); edema. One month later: CT: substantial resolution of the lesions while the patient was receiving only corticosteroid therapy (contrast). Nine months later: MRI: after treatment with both antibiotics and corticosteroids, demonstrated resolution of the lesions, except for a subtle abnormality in the left midbrain (Tl + contrast).	[50]
46	Lymphoplasmacytic infiltrate with extensive perivascular inflammation.		MRI: dural thickening in the area of the lesion. CT: isolated, peripherally located, contrast-enhancing lesion of the brain.	[51]
47			CT: isolated, peripherally located, contrast-enhancing lesion of the brain.	[51]
48		“Increased lymphocytes, elevated protein, and decreased glucose.” Second lumbar puncture: FTA-ABS: positive.	MRI: mass; signal intensity isointense to cortex on (T1 + double spin echo); enhanced markedly (T1, contrast). CT: suprasellar enhancing mass.	[52]
49			MRI: intense enhancement	[53]
50		Erythrocytes: 6; lymphocytes: 0; glucose level: 41; protein level: 83; culture: negative; VDRL: positive (units are not reported).	MRI: normal.	[54]

## Data Availability

The collected data and references are listed in this case report.

## Search Criteria

The authors identified references using a search of the EMBASE, Google Scholar, and Medline (PubMed) databases for English language-only articles published between 1989 and December 2019 using the following terms: “Syphilis,” “*Treponema pallidum*,” “Syphilis MSM,” “Syphilis men who have sex with men,” “*Treponema pallidum* central nervous system,” “Syphilis Western countries,” “Epidemiology syphilis,” ‘Epidemiology *Treponema pallidum*,” “Transmitted sexual diseases,” “STD,” “STI,” “*Treponema pallidum* brain,” “*Treponema pallidum* CNS,” “*Treponema pallidum* head,” “Gumma syphilis,” “Gumma central nervous system,” “Gumma CNS,” “Gumma brain,” “Gumma intramedullary,” “Syphilis tumour,” and “Syphilis tumor.” The authors also reviewed the websites of the United States Centers for Disease Control and Prevention, the European Centre for Disease Prevention and Control, the Robert Koch Institute (German federal government agency and research institute responsible for disease control and prevention), and UpToDate, Inc. (Wolters Kluwer, Alphen aan den Rijn, Netherlands). The databases were analyzed by two authors independently of one another using these search criteria.
